# A stacked ensemble model with NNLS-based weighting for influenza forecasting: a case study of Anhui Province, China

**DOI:** 10.3389/fpubh.2026.1806095

**Published:** 2026-05-05

**Authors:** Qingqing Zhu, Minglei Zhu, Yuhang Cai, Junwei Xiang, Shuwen Li, Biao Zhu, Meng Zhu, Lei Gong, Sai Hou, Jun He, Junling Yu, Jiabing Wu

**Affiliations:** 1 Anhui Center of Disease Control and Prevention, Hefei, China; 2 School of Public Health, Anhui Medical University, Hefei, China; 3 College and Hospital of Stomatology, Anhui Medical University, Key Lab. of Oral Diseases Research of Anhui Province, Hefei, China

**Keywords:** ARIMA, ensemble learning, influenza forecasting, influenza surveillance, Prophet, stacking (NNLS), time series modeling, XGBoost

## Abstract

**Background:**

Influenza poses a significant global public health threat, with its pandemic potential and seasonal variability presenting formidable challenges to prediction accuracy. This study leverages high-quality weekly data (incidence rates, viral subtypes, and meteorological indicators) from the provincial influenza surveillance system in Anhui Province, eastern China, spanning 2015–2025. A multi-source data fusion model was developed to overcome the limitations of traditional methods in modeling nonlinear transmission dynamics and multi-factor synergistic effects.

**Methods:**

Single models were constructed using ARIMA, Prophet, and XGBoost, then stacked into an interpretable ensemble model (Stacked-NNLS) using non-negative least squares (NNLS). Performance was comprehensively evaluated using *R*^2^ (explained variance), RMSE (root mean square error), MAE (mean absolute error), and MAPE (mean absolute percentage error).

**Results:**

ARIMA exhibits poor fit for non-stationary sequences (training set *R*^2^ = −3.66; test set *R*^2^ = 0.03). Prophet effectively captures long-term trends (training/test set *R*^2^ = 0.38/0.88). XGBoost shows overfitting (training/test set *R*^2^ = 0.99/0.74). The Stacked-NNLS model demonstrated significantly superior robustness (training/test *R*^2^ = 0.94/0.94), outperforming baseline models across all metrics.

**Conclusion:**

By integrating statistical, seasonal, and nonlinear modeling approaches, Stacked-NNLS demonstrated robust predictive performance in capturing influenza trends, seasonal fluctuations, and complex interactions among multiple factors, suggesting its potential utility for infectious disease early warning and public health decision-making.

## 1 Introduction

Influenza, as an acute respiratory infectious disease capable of causing global pandemics, has made epidemic forecasting a major focus of public health research (1). In recent years, with the rapid advancement of information technology and data science, an increasing number of countries and regions have begun integrating data-driven modeling approaches into influenza surveillance systems to enhance the scientific rigor and timeliness of epidemic prevention and control (2–4). At the international level, the U. S. Centers for Disease Control and Prevention (CDC) pioneered influenza trend forecasting based on ILINet data by combining traditional statistical methods with machine learning models for weekly epidemic assessment and risk alerts (5). Google previously launched the “Google Flu Trends” project, which attempted to predict influenza incidence on the basis of internet users’ search behavior. Although it initially demonstrated some predictive capability, the model suffered from limited accuracy and was unable to adequately account for data noise and external influences, ultimately leading to its discontinuation (6). The European Influenza Surveillance Network (EISN) primarily relies on historical case data for time-series extrapolation. While this modeling approach has some value under relatively stable conditions, it responds slowly to sudden outbreaks or disruptive factors, resulting in limited early warning capability (7). In recent years, Japan has actively explored influenza forecasting methods based on Bayesian statistics and deep learning frameworks, thereby gradually establishing a corresponding technical framework. However, these approaches have rarely incorporated meteorological factors, social activity patterns, holidays, or other intervention-related variables, resulting in limited adaptability to dynamic changes (8).

Influenza modeling research in China started relatively late and has largely focused on the optimization and extension of traditional ARIMA models (9, 10). Some studies have explored emerging models such as Prophet and Long Short-Term Memory (LSTM) networks, with moderate success in short-term forecasting (11, 12). Existing domestic research predominantly relies on single-model approaches, lacks multi-model integration, and remains limited in its integration of high-frequency meteorological data with influenza virus subtype data. Many model designs fail to adequately account for nonlinearity and structural noise in the data, resulting in poor stability, limited interpretability, and a high risk of overfitting. This limits their reliable application in public health practice (13, 14).

In response to these gaps, this study used nearly a decade of high-quality influenza surveillance data from Anhui Province in eastern China and integrated key variables, including weekly meteorological indicators from the provincial meteorological bureau and positivity rates of multiple influenza virus strains from the provincial respiratory surveillance network laboratories. Three representative forecasting models, ARIMA, Prophet, and XGBoost, were constructed and compared, and a weighted stacking ensemble approach was applied to integrate their complementary strengths. By combining surveillance, meteorological, and virological data, this framework was designed to address limitations in generalization and overfitting associated with individual models. Therefore, the aim of this study was to develop and evaluate a stacked ensemble forecasting framework for influenza incidence in Anhui Province and to explore its potential value for influenza early warning and public health decision-making.

## 2 Materials and methods

**Figure 1:**
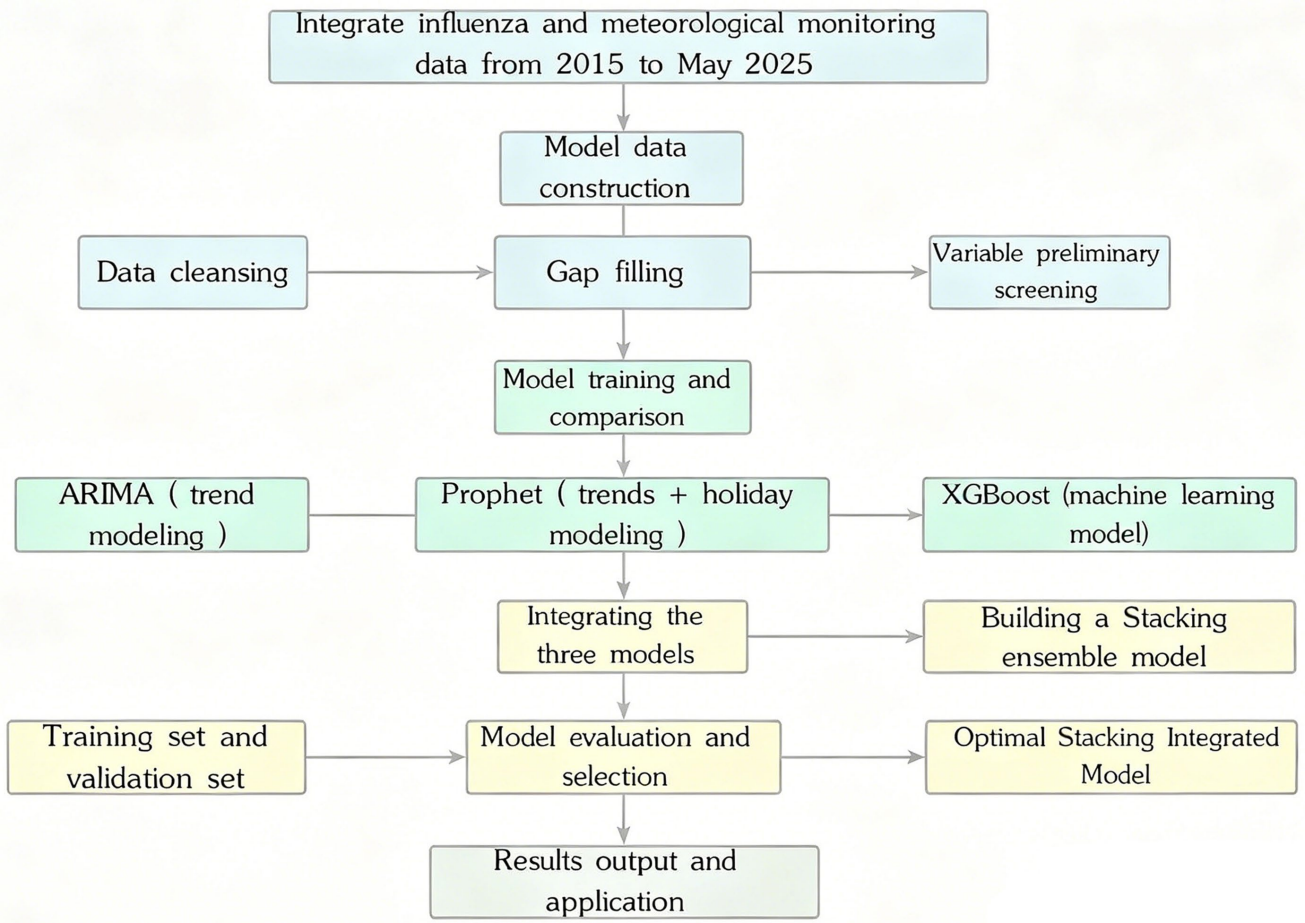
Overall workflow of data integration, preprocessing, model development, evaluation, and forecasting in this study.

The overall workflow of the study is shown in Figure 1.

### 2.1 Data sources

Data for this study were obtained from the China Influenza Surveillance Information System (CISIS). We extracted influenza surveillance records for Anhui Province from 2015 to 2025, including incidence rates, laboratory-confirmed positivity rates, and viral serotypes. Additionally, daily meteorological data for the 16 prefecture-level cities of Anhui Province from 2016 to 2023 were obtained from the Anhui Provincial Meteorological Bureau, including mean, maximum, and minimum temperatures; mean relative humidity; mean 2-min wind speed; and precipitation measured by a rain gauge from 20:00 to 20:00.

### 2.2 Data processing and modeling framework

Influenza surveillance data from Anhui Province spanning May 2015 to May 2025 were integrated with selected meteorological data, and duplicate records, key-value conflicts, and missing values were systematically addressed. The dataset was partitioned into a training set (August 2015 to August 2024) and a test set (September 2024 to May 2025). Missing values were handled according to variable type. Missing values in the influenza incidence series were imputed using exponential moving average (EMA) and Kalman filtering, whereas missing meteorological values were imputed using linear interpolation. The effects of imputation were evaluated by comparing data distribution plots before and after processing. An interpretable decision-tree-based approach was further used to assess the validity of the imputed values. A detailed summary of missing-data proportions is provided in Supplementary Table S1.

Influenza activity during 2020 to 2022 remained at near-zero levels for extended periods, largely due to non-pharmaceutical interventions during the COVID-19 pandemic. These observations were retained in the training set and were not treated as outliers. We regarded this period as a meaningful epidemiological phase rather than a data anomaly. Missing values occurring during this interval were processed using the same rules as those used for the full dataset. This strategy preserved the continuity of the time series and enabled the model to learn low-activity patterns under extreme intervention conditions.

Based on the processed dataset, an ensemble prediction framework was constructed by integrating three base learners: the Autoregressive Integrated Moving Average (ARIMA) model, the Prophet additive time series model, and the eXtreme Gradient Boosting (XGBoost) model. The performance and parameter settings of the individual models were compared, and their predictions were ultimately integrated using a stacking strategy to develop a comprehensive model for predicting influenza incidence in Anhui Province.

### 2.3 Model specification and parameter optimization

Differentiated parameter optimization strategies were used to ensure comparability and reproducibility across models. For the ARIMA model, the optimal orders *(p, d, q)(P, D, Q)_s* were identified automatically using the corrected Akaike information criterion (AICc), with the seasonal period *s* set to 52 weeks to reflect the annual periodicity of influenza surveillance data. This approach reduced bias associated with subjective parameter selection.

For the Prophet model, yearly and weekly seasonality were enabled to capture long-term trends and within-week variation in influenza activity, while daily seasonality was disabled. Holiday effects were incorporated as external regressors using a holiday flag and a holiday count variable to quantify the impact of statutory holidays, such as the Spring Festival, on case reporting.

For the XGBoost model, hyperparameter tuning was performed using random search with 10-fold cross-validation to reduce overfitting and enhance generalizability. The search space included tree depth, learning rate, number of trees, and feature subsampling. Specifically, tree depth ranged from 1 to 10, learning rate from 0.001 to 0.1, number of trees from 50 to 100, and 1 to 5 features were randomly sampled for each tree. The final hyperparameter combination was selected through a composite assessment of RMSE, MAE, and MAPE, balancing predictive accuracy and model stability.

For the stacked ensemble model, coefficients were optimized using non-negative least squares (NNLS). The coefficients were constrained to be non-negative and were estimated by fitting the training-set predictions to the observed values, thereby ensuring that coefficient estimation was independent of the test set and avoiding information leakage. These NNLS coefficients were not constrained to sum to one. They therefore represent non-negative combination coefficients rather than normalized averaging weights.

### 2.4 Evaluation metrics

Model performance was evaluated using root mean square error (RMSE), mean absolute error (MAE), mean absolute percentage error (MAPE), coefficient of determination (*R*^2^), and adjusted *R*^2^. The definitions and interpretations of these metrics are presented in Table 1.

**Table 1:** Definitions and interpretations of the evaluation metrics.

Metric	Definition and interpretation
RMSE	Reflects the average magnitude of deviation between predicted and observed values and is more sensitive to large errors. It is expressed in the same unit as the original case counts.
MAE	Represents the average absolute difference between predicted and observed values. Compared with RMSE, it is more robust and less affected by extreme prediction errors.
MAPE	A relative error metric that facilitates comparisons across regions or disease categories. It may become unstable when case counts are close to zero.
*R* ^2^	Indicates the proportion of variance explained by the model. Negative values indicate that the model performs worse than a simple mean-based prediction.
Adjusted *R*^2^	A modified form of *R*^2^ that accounts for sample size and the number of model parameters. It is more appropriate for comparing models with different levels of complexity.

## 3 Results

### 3.1 Feature engineering and variable selection

**Figure 2:**
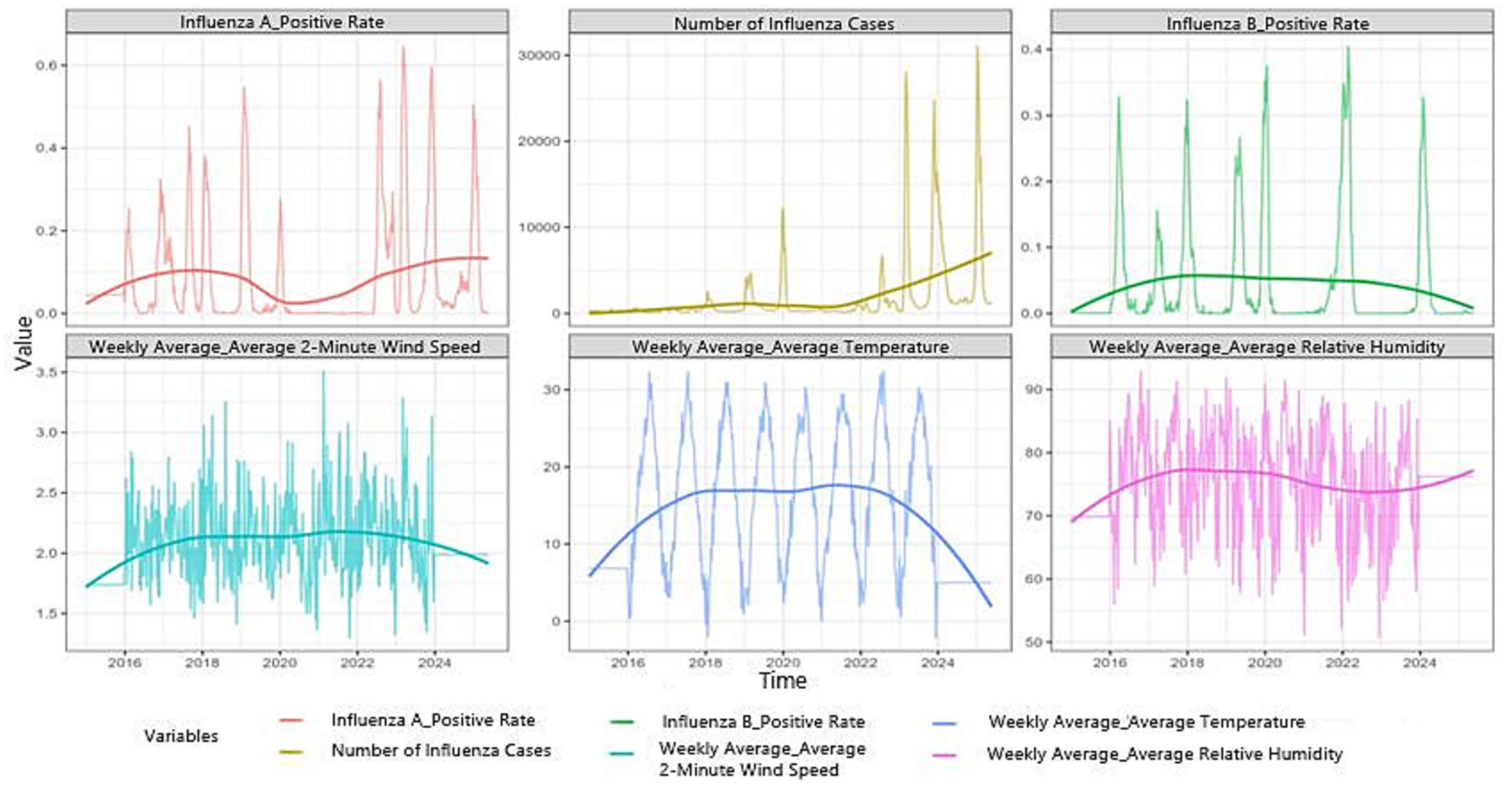
Temporal patterns of influenza incidence, viral positivity rates, and meteorological variables in Anhui Province during the study period.

Time-series decomposition and exploratory analysis revealed clear annual cyclicality and medium- to long-term trends in influenza incidence, together with substantial residual fluctuations during several epidemic periods (Supplementary Figures S1, S2). After feature engineering and variable screening, the final model incorporated key epidemiological and meteorological variables, including viral positivity rates, temperature, humidity, and wind speed. Figure 2 illustrates the temporal patterns and distributional characteristics of the main variables. Influenza activity showed pronounced seasonality, with higher levels during colder periods, whereas temperature exhibited the strongest seasonal variation among the meteorological factors. Influenza A activity increased slightly from 2022 to 2025, whereas influenza B activity remained relatively stable or declined slightly.

### 3.2 Training and test-set performance of the candidate models

**Figure 3:**
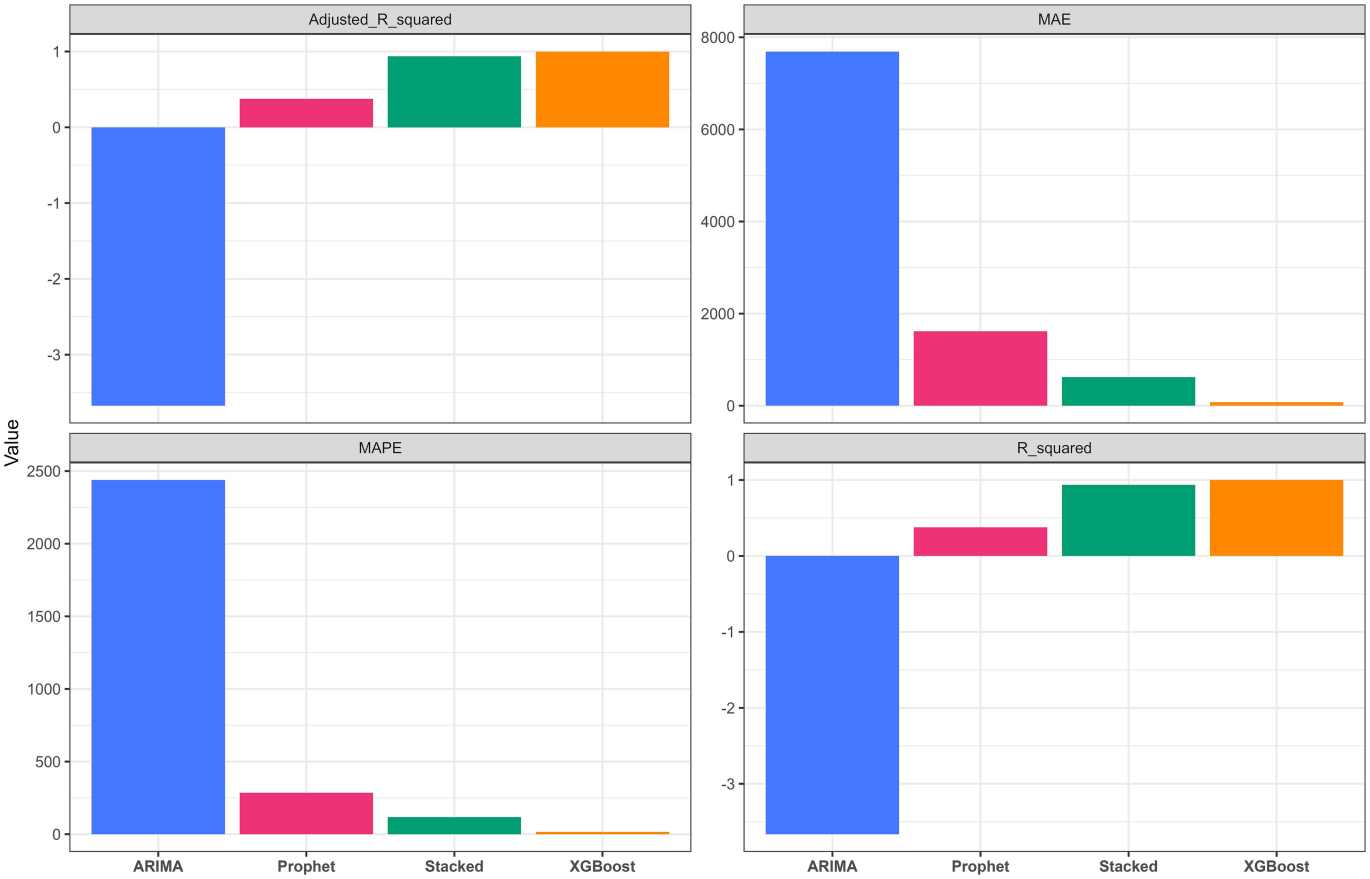
Comparison of model performance metrics on the training set across ARIMA, Prophet, XGBoost, and the stacked ensemble model. Metrics include *R*^2^, MAE, MAPE, and adjusted *R*^2^.

**Figure 4:**
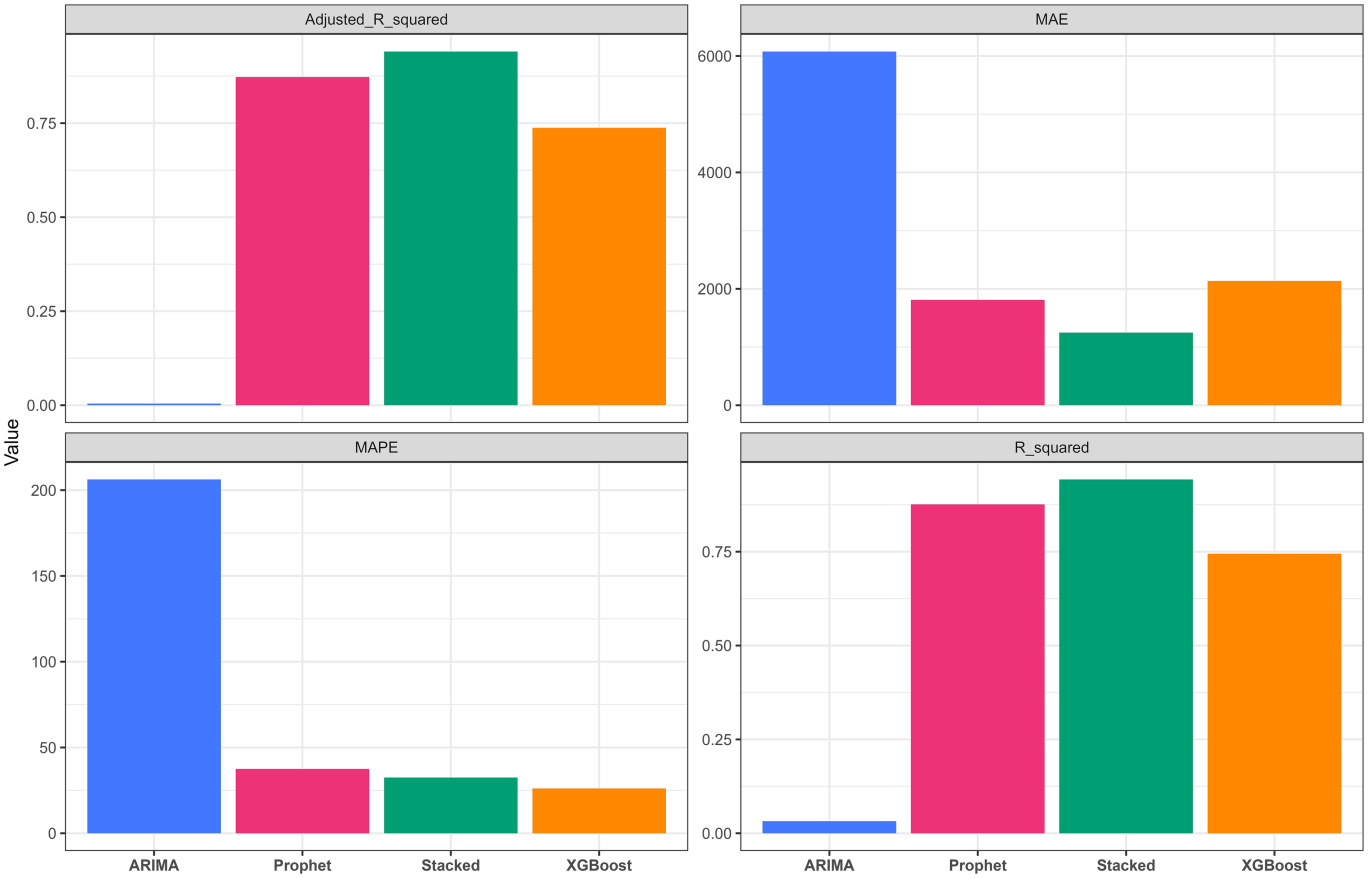
Comparison of model performance metrics on the test set across ARIMA, Prophet, XGBoost, and the stacked ensemble model. Metrics include *R*^2^, MAE, MAPE, and adjusted *R*^2^.

The comparative performance of the candidate models on the training and test sets is summarized in Figures 3, 4 and Table 2. On the training set, the ARIMA model performed poorly, yielding a negative *R*^2^ (−3.66), whereas the Prophet model showed moderate fit (*R*^2^ = 0.38). Both XGBoost and the stacked ensemble model achieved training-set *R*^2^ values above 0.90, indicating strong in-sample fitting performance (Figure 3). Visual comparisons of the fitted trajectories are provided in Supplementary Figures S3–S6.

**Table 2:** Test set model performance metrics.

Model	RMSE	MAE	MAPE	*R* ^2^	Adjusted *R*^2^
ARIMA	8343.74	6076.40	206.17	0.03	0.004
Prophet	2983.91	1809.31	37.52	0.88	0.87
XGBoost	4284.90	2133.83	26.14	0.74	0.74
Stacked	2041.72	1245.72	32.46	0.94	0.94

**Figure 5:**
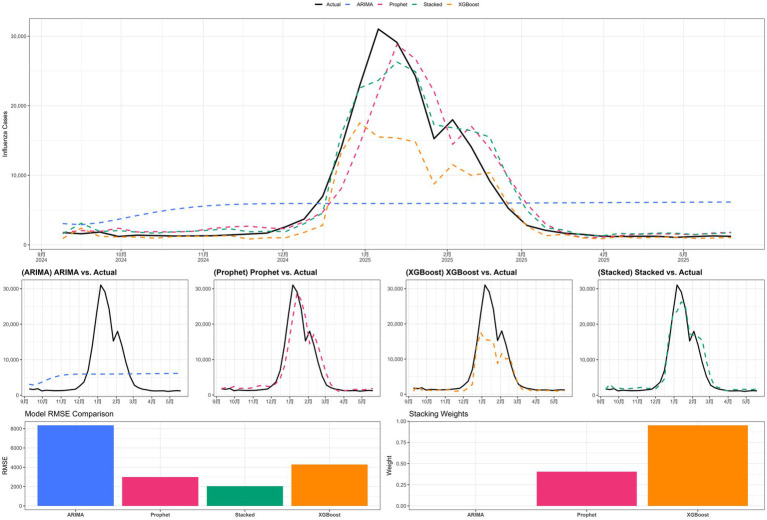
Predicted versus observed influenza incidence on the test set across candidate models, together with RMSE comparison and the NNLS coefficients of the stacked ensemble.

On the test set, the stacked ensemble model achieved the best overall performance, with the highest *R*^2^ (0.94) and the lowest error metrics among the candidate models (Figure 4 and Table 2). The Prophet model also performed well (*R*^2^ = 0.88), whereas XGBoost showed a decline in predictive performance relative to the training set (*R*^2^ = 0.74), suggesting possible overfitting. ARIMA exhibited the weakest predictive performance (*R*^2^ = 0.03). As shown in Figure 5, the stacked ensemble model tracked the observed epidemic trajectory more closely than the individual models, particularly during the major winter peak and the subsequent decline. Detailed performance metrics for the training set are provided in Supplementary Table S2.

The NNLS procedure assigned a coefficient of 0 to ARIMA, whereas Prophet and XGBoost received coefficients of 0.405 and 0.952, respectively. This result was consistent with the poor training-set fit of ARIMA (*R*^2^ = −3.66), suggesting that its linear autoregressive structure was not well suited to the non-stationary and structurally disrupted epidemic series analyzed in this study. By contrast, Prophet and XGBoost contributed more substantially to the ensemble under the present data conditions.

### 3.3 Interpretation of the stacked ensemble model

**Figure 6:**
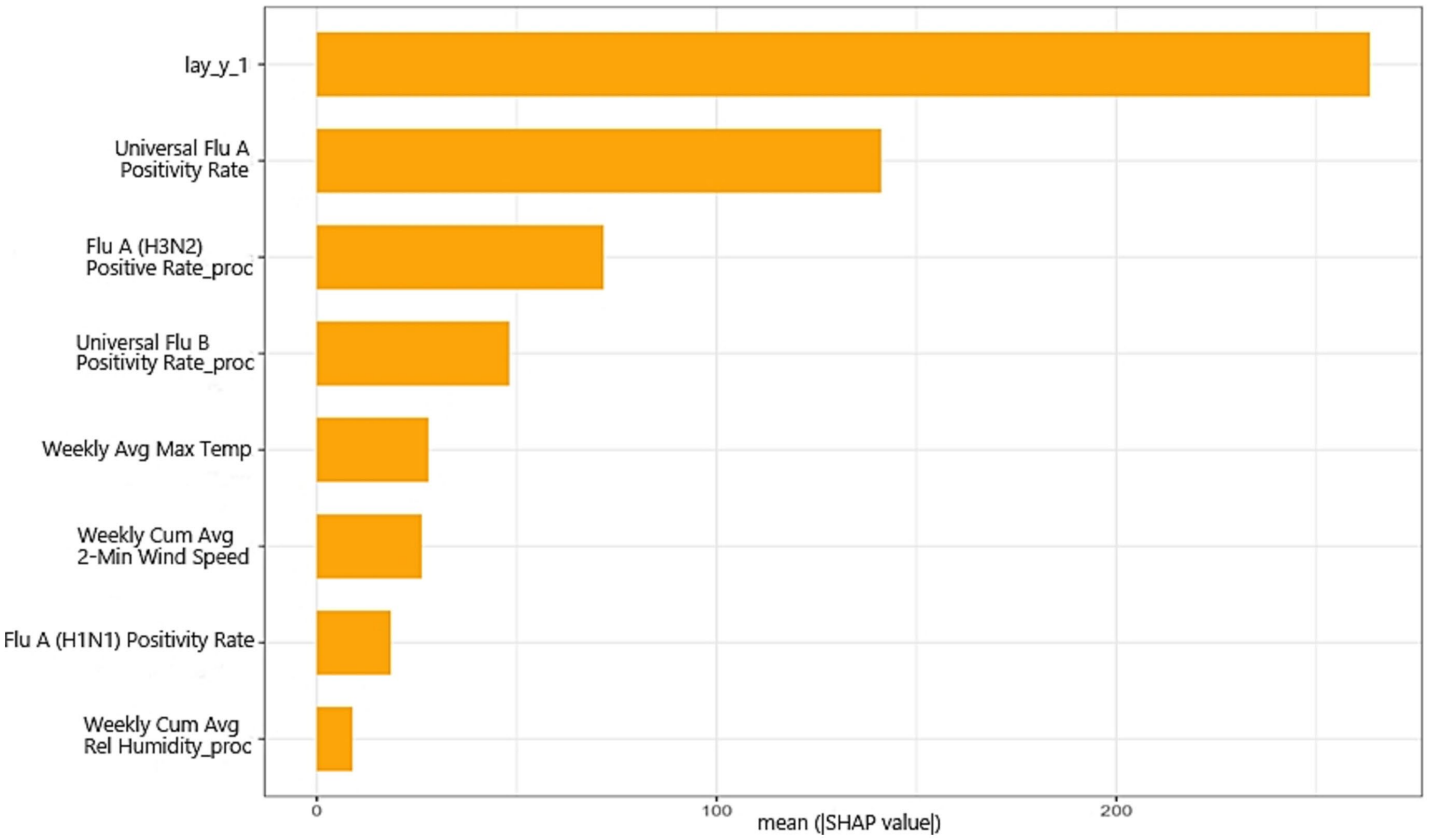
Global feature importance of the stacked ensemble model based on mean absolute SHAP values. Variables with the suffix “proc” were transformed using Box–Cox transformation and standardization.

The SHAP analysis showed that the previous-week case count was the most influential predictor in the stacked ensemble model, with a mean absolute SHAP value of approximately 263, followed by influenza A positivity rate (approximately 141) and the positivity rates of specific influenza subtypes, including H3N2 (approximately 71) and influenza B (approximately 48) (Figure 6). Meteorological variables, particularly weekly average maximum temperature (approximately 28) and weekly cumulative average 2-min wind speed (approximately 26), also contributed to the predictions, although to a lesser extent. Overall, these findings suggest that the model was driven primarily by short-term temporal dependence and virological indicators, with meteorological variables acting as supplementary modifiers. More detailed SHAP dependence patterns and decision-path visualizations are presented in Supplementary Figures S7, S8.

### 3.4 Six-week forecasting of influenza incidence in Anhui Province

**Figure 7:**
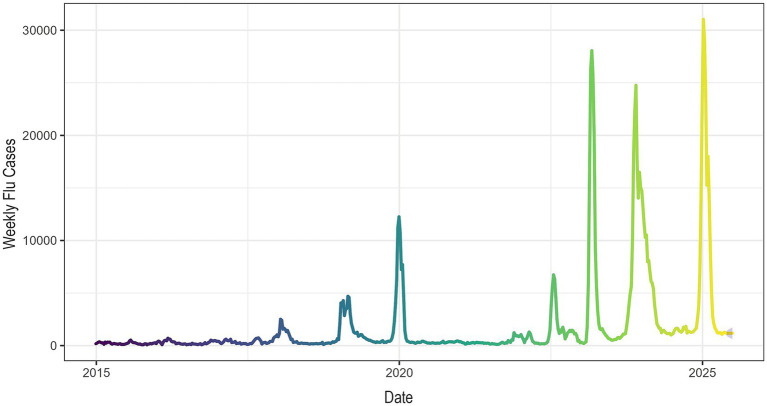
Six-week forecast of influenza incidence in Anhui Province generated by the stacked ensemble model, showing the projected short-term trend after the end of the observed series.

The stacked ensemble model was further used to generate six-week forecasts of influenza incidence in Anhui Province (Figure 7 and Table 3). The forecasts suggested that influenza activity would remain relatively stable over the short term, with central estimates remaining around 1,100–1,200 cases per week and no clear sustained upward or downward trend. Prediction intervals widened gradually over the forecast horizon, reflecting increasing uncertainty over time. In the first 1–2 weeks, the forecast intervals remained relatively narrow, with lower and upper bounds of approximately 900 and 1,300–1,400 cases, respectively. By the fifth and sixth weeks, the interval had widened to approximately 600–700 cases at the lower bound and 1,600–1,700 cases at the upper bound.

**Table 3:** Projected number of influenza cases over the next 6 weeks.

Date	Number	Lower	Upper	Actual
2025/5/26	1,162	943	1,381	1,247
2025/6/2	1,162	852	1,471	1,095
2025/6/9	1,162	783	1,541	1,057
2025/6/16	1,162	724	1,599	1,109
2025/6/23	1,162	673	1,651	1,101
2025/6/30	1,162	626	1,698	908

Despite this increase in uncertainty, the forecast intervals remained consistent with recent observed values. For example, the observed value at the end of May 2025 was 1,247 cases, which was close to the central forecast range of 1,100–1,200 cases, whereas the observed value at the end of June 2025 had declined to 908 cases but still remained within the predicted interval. The model also captured the broader historical pattern of seasonal peaks and short-duration surges, suggesting that it was able to preserve the dominant epidemic structure while generating short-term projections. An enlarged view of the short-term forecast is provided in Supplementary Figure S9.

## 4 Discussion

### 4.1 Predictive performance and public health implications

The forecasting framework developed in this study demonstrated robust short- to medium-term predictive capability, with the stacked ensemble model achieving the best overall performance on the test set. This finding has important implications for public health decision-making. By anticipating potential epidemic trajectories several weeks in advance, health authorities may be better positioned to strengthen surveillance, support preparedness planning, and allocate resources in a more timely manner. Measures such as strengthening surveillance, stockpiling antiviral medications and medical supplies, expanding hospital capacity and workforce preparedness, and conducting health education and vaccination outreach for high-risk populations may help reduce the burden during periods of increased influenza activity. Tiered response systems may also be adjusted dynamically and in a timely manner to match anticipated epidemic conditions (15). Previous research has shown that accurate outbreak forecasting can improve the effectiveness of public health responses, for example, by facilitating advance preparation for increases in hospitalizations, thereby reducing the burden on both society and healthcare systems during influenza outbreaks (16).

### 4.2 Complementary strengths of different models and the value of ensemble learning

This forecasting framework integrated ARIMA, Prophet, and XGBoost models to exploit the complementary strengths of different modeling strategies. The ARIMA and Prophet models were particularly useful for capturing linear trends and seasonal cycles in the time series, whereas XGBoost was better suited to characterizing nonlinear relationships and interactions among predictors. No single model can perform optimally across all tasks, whereas ensemble learning allows the strengths of different models to be combined. In this study, the stacking approach improved predictive robustness by integrating the advantages of statistical time-series modeling and machine learning.

Previous studies have shown that a single model is unlikely to capture all patterns and characteristics of infectious disease time series, whereas ensemble approaches can improve forecasting performance by combining the strengths of different models (17). Reports from other regions have similarly shown that multi-model ensembles often outperform single-model influenza forecasts (18). Taken together, these findings highlight the value of ensemble learning for capturing the multifaceted dynamics of epidemic trends and support the rationale for using a stacked framework in influenza forecasting.

### 4.3 Comparative model performance and generalization of the stacked ensemble

Among the candidate models, the stacked ensemble model demonstrated the strongest generalization performance. On the test set, it achieved the highest *R*^2^ (0.94), outperforming Prophet (0.88), XGBoost (0.74), and ARIMA (0.03), while also yielding the lowest overall error metrics. Although both XGBoost and the stacked ensemble model showed strong in-sample fitting performance, their test-set behavior differed substantially, with XGBoost showing a noticeable decline in predictive accuracy, suggesting possible overfitting.

This pattern likely reflects the ability of the stacked ensemble to balance the bias and variance of different learners. As a high-complexity tree-based model, XGBoost is more prone to capturing noise and period-specific irregularities, particularly when the epidemic series contains structural disruptions such as those observed during the COVID-19 period. By contrast, the stacked ensemble appears to have benefited from an implicit regularization effect. The trend and seasonal information captured by Prophet, together with the linear extrapolation provided by ARIMA, may have offset some of the more extreme predictions generated by XGBoost, thereby improving robustness and preserving predictive continuity during anomalous periods.

These observations are consistent with previous studies showing that ensemble methods can significantly improve influenza forecasting accuracy. For example, one study reported that a stacked ensemble reduced RMSE by approximately 24% relative to XGBoost alone, while another study in Hong Kong found that an adaptive weighted ensemble achieved an 8-week forecast RMSE that was 52% lower than that of baseline models, outperforming individual models across epidemic phases including growth, plateau, and decline (17, 18). Taken together, the present findings suggest that the stacked ensemble model achieved better overall predictive performance than the individual models evaluated in this study, while showing stronger generalization on the test set. From a practical perspective, additional strategies such as dynamic weight adjustment may further improve robustness, particularly during extreme anomalous periods.

It is also noteworthy that the NNLS procedure assigned a coefficient of 0 to ARIMA in the final ensemble. Rather than removing ARIMA from the candidate pool, we retained it as a classical statistical baseline representing a linear autoregressive assumption. This design allowed the ensemble framework to evaluate, in a transparent and data-driven manner, whether such a component contributed useful predictive information. In the present study, the zero coefficient indicated that ARIMA provided little additional value for the final prediction, while the NNLS optimization prevented this underperforming candidate from affecting the ensemble output.

### 4.4 Model interpretability, transparency, and cross-departmental communication

An important advantage of the stacked ensemble framework is that its predictions remained interpretable despite its higher predictive complexity. Complex machine learning and ensemble models are often perceived as “black boxes,” which can undermine trust in model outputs among public health decision-makers. In this study, SHAP analysis helped clarify the relative importance of the main predictors and showed that the model was driven primarily by the previous-week case count, influenza A positivity rate, and subtype-specific virological indicators, with meteorological variables such as temperature and wind speed making secondary contributions. These results are epidemiologically plausible and enhance confidence in the model’s internal logic. More broadly, SHAP-based interpretation has been increasingly used to explain the contribution of individual predictors in complex models and to improve transparency in health-related prediction tasks (19).

Improved interpretability is also important for communication across sectors. By translating complex algorithmic outputs into epidemiologically meaningful signals, interpretable models can facilitate consensus-building among health departments, meteorological agencies, and hospital administrators. For example, when the model indicates that low temperature and increased virological activity are jointly associated with rising future case counts, decision-makers can more readily justify targeted interventions such as intensified cold-season prevention campaigns or more focused vaccination outreach. In this sense, interpretability does not merely improve technical transparency; it also strengthens the practical usability of the forecasting system in real-world prevention and control settings.

### 4.5 Six-week forecasts and their early warning implications

The six-week forecasts generated by the stacked ensemble model suggest that influenza activity in Anhui Province is likely to remain in a relatively stable plateau phase over the short term, with central estimates remaining around 1,100–1,200 cases per week. This finding has important implications for early warning and response planning. On the one hand, a sustained weekly case count at this level suggests continued influenza activity, indicating that public health authorities should maintain enhanced surveillance and existing prevention measures, including continuous virological surveillance, case reporting, and protective measures in high-risk settings. On the other hand, the prediction intervals widened progressively as the forecast horizon extended, indicating increasing uncertainty over time.

Although the central forecast did not indicate a sharp upward or downward shift, the upper bound of the prediction interval still captured plausible higher-risk scenarios. In the first 1–2 weeks, the predicted range remained relatively narrow, but by weeks five to six the interval had widened substantially, reflecting reduced precision at longer lead times. This pattern is consistent with the expected behavior of short-term forecasting models and highlights the importance of interpreting point forecasts together with uncertainty bounds. At this level of precision, the forecast may provide useful support for short-term early warning and preparedness planning, but it may be less suitable as a standalone basis for triggering formal emergency responses, especially at longer lead times. If observed case counts begin to approach the upper bound of the interval, this may warrant enhanced monitoring and preparedness planning. Conversely, if observed counts remain close to the lower bound and stay below historical levels, routine surveillance and continued monitoring may be sufficient.

Overall, the six-week forecasts provide a useful window for preparedness and response. Early warning before a potential increase in transmission may allow interventions to be implemented sooner, while also helping to avoid unnecessary overreaction when influenza activity is likely to remain stable or decline. At the same time, the forecast interval should be interpreted as reference information for short-term risk assessment rather than as a fixed operational threshold on its own. In practice, formal response thresholds would still need to be determined in combination with local surveillance practice, healthcare capacity, and historical epidemic patterns. Incorporating forecast information into public health decision-making may therefore support more timely and proportionate responses.

### 4.6 Implications of the COVID-19 pandemic for influenza modeling

The COVID-19 pandemic introduced substantial challenges for influenza forecasting by altering both transmission dynamics and the quality of surveillance data. During 2020–2022, large-scale non-pharmaceutical interventions (NPIs), including social distancing, mask-wearing, school and workplace closures, and travel restrictions, substantially suppressed influenza transmission and led to historically anomalous declines in influenza activity. Studies have shown that influenza transmission declined sharply after emergency public health measures were implemented in multiple settings, including the United States and Singapore, and comparable declines were also reported in Europe, South Korea, and other regions (20). For forecasting models that rely heavily on historical seasonal regularity, these shifts created a major challenge because near-zero influenza activity had rarely been observed previously and therefore provided few meaningful precedents for model learning.

At the same time, the pandemic affected the continuity and representativeness of influenza surveillance data. The diversion of healthcare resources to COVID-19 care, interruptions in sentinel surveillance, and changes in healthcare-seeking behavior likely contributed to underreporting, discontinuities, and reduced comparability in surveillance data. Similar concerns were reflected in surveillance changes elsewhere. For example, the CDC shifted its influenza forecasting metric from outpatient influenza-like illness (ILI) rates to hospitalization counts beginning in 2021 because altered outpatient visit patterns reduced the interpretability of ILI data. Together, these factors weakened the reliability of historical seasonal patterns and complicated model training during anomalous periods.

These disruptions were not only epidemiological but also methodological. For models such as those used in this study, directly incorporating pandemic-period data may introduce noise and bias, whereas excluding them entirely may remove potentially informative recent patterns. This highlights the importance of robustness during anomalous periods. Possible strategies include separately modeling periods affected by major interventions, incorporating covariates that reflect intervention intensity, or applying adaptive updating approaches to reduce the influence of transient irregularities.

Encouragingly, some ensemble models have continued to show good adaptability under post-pandemic conditions. For instance, one study found that, when post-pandemic data from 2023 to 2024 were used as a test set, an adaptive weighted ensemble model reduced RMSE by 39% compared with an individual model (18). Nevertheless, recent work by the U. S. FluSight team showed that even multi-model ensemble forecasts could lose accuracy during rapid post-pandemic resurgences and sudden epidemic peaks (21). Overall, the COVID-19 pandemic highlighted the limitations of influenza forecasting under extreme conditions while also emphasizing the need for more robust updating strategies and anomaly-aware modeling frameworks for future public health emergencies.

### 4.7 Limitations

Several limitations should be noted. First, although the present study compared representative statistical and machine learning models, it did not include recent deep learning architectures such as LSTM- or Transformer-based models. Therefore, the comparative scope of the study remains incomplete, and caution is needed when interpreting the methodological advantage of the proposed framework. Second, the influenza surveillance series included an anomalous period during the COVID-19 pandemic, when large-scale non-pharmaceutical interventions substantially altered influenza transmission patterns and may also have affected surveillance continuity and representativeness. Although these observations were retained because they reflected a meaningful epidemiological phase, they may still limit the stability and generalizability of the model under changing post-pandemic conditions. Third, the modeling strategy in this study prioritized interpretability, computational efficiency, and practical applicability in routine public health settings. Future studies may extend the current framework by incorporating deep learning models and by examining whether broader or hybrid ensemble strategies can further improve forecasting performance.

## 5 Conclusion

In conclusion, the stacked ensemble model integrated the strengths of statistical, seasonal, and nonlinear modeling approaches and achieved better predictive performance than the individual models evaluated in this study. It captured influenza incidence trends, seasonal fluctuations, and interactions among multiple determinants, and showed potential utility for short- to medium-term influenza forecasting. These findings suggest that the proposed framework may provide useful support for early warning, resource planning, and evidence-based public health decision-making in influenza prevention and control in Anhui Province.

## Data Availability

The data analyzed in this study is subject to the following licenses/restrictions: the datasets generated for this study are not publicly available due to ethical and legal considerations. Requests to access the data may be considered by the corresponding authors on a case-by-case basis, and the authors reserve the right to decline data sharing where appropriate. Requests to access these datasets should be directed to QZ, zhuqingqing@ahmu.edu.cn.
