# Supplementary material for: A stacked ensemble model with NNLS-based weighting for influenza forecasting: a case study of Anhui Province, China
Source: Front Public Health. 2026 May 5;14:1806095. doi: 10.3389/fpubh.2026.1806095 (PMC13183840; doi:10.3389/fpubh.2026.1806095)
Supplement: Supplementary file 1 [file Data_Sheet_1.PDF]

Supplementary Material

Supplementary Figures

Figure S1. STL decomposition of the influenza incidence time series.
Figure S2. STL decomposition of the first principal component derived from PCA.
Figure S3. Fitting performance of the ARIMA model for influenza incidence.
Figure S4. Fitting performance of the Prophet model for influenza incidence.
Figure S5. Fitting performance of the XGBoost model for influenza incidence.
Figure S6. Fitting performance of the stacked ensemble model for influenza incidence.
Figure S7. SHAP dependence plots for key features in the stacked ensemble model.
Figure S8. Decision paths of feature contributions in the stacked ensemble model.
Figure S9. Enlarged view of the six-week forecast of influenza incidence.

Supplementary Tables

Table S1. Missingness proportion for study variables.
Table S2. Performance metrics of the candidate models on the training set.

Supplementary Notes

Note S1. Variable preprocessing.

Variables with the suffix “proc” underwent Box–Cox transformation and standardization before model fitting.

Note S2. Additional visualizations.

Supplementary Figures S3–S8 provide additional visual evidence supporting the comparative fitting performance and interpretability of the candidate models.

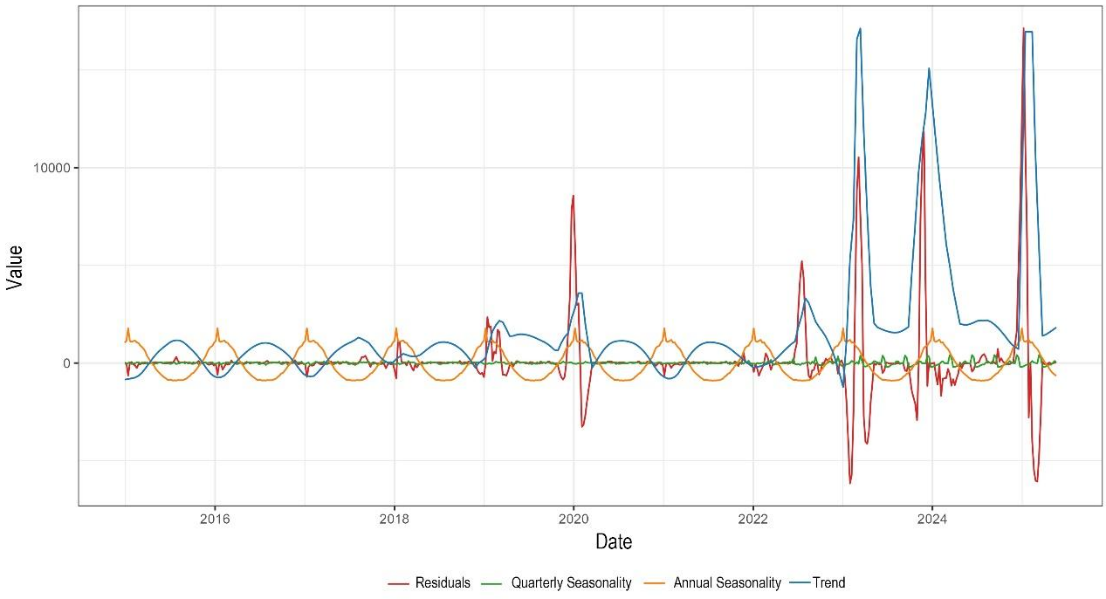

Figure S1. STL decomposition of the influenza incidence time series.

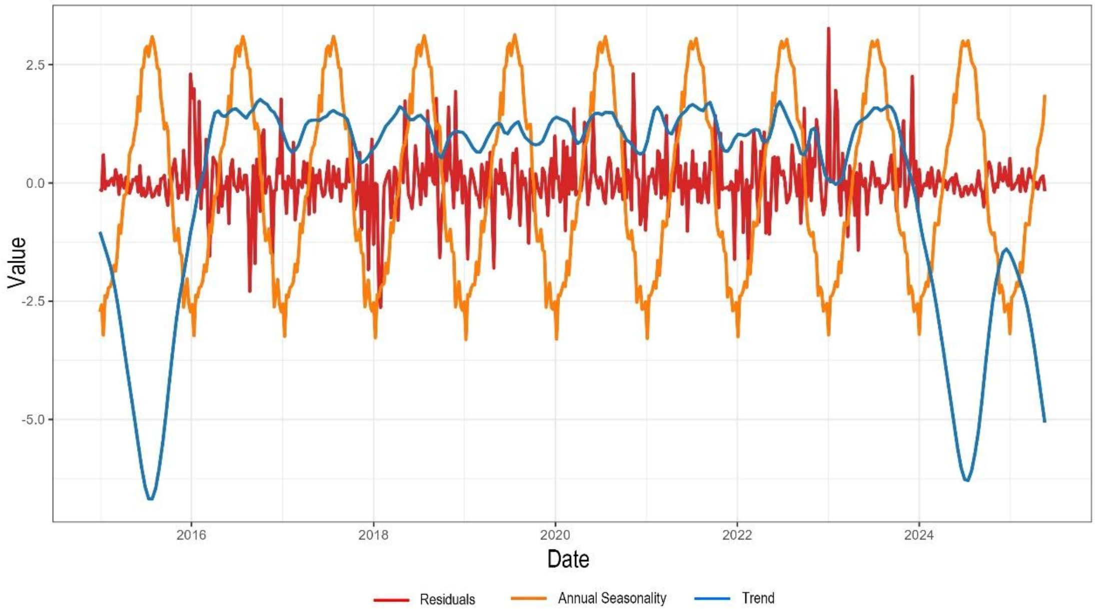

Figure S2. STL decomposition of the first principal component derived from PCA.

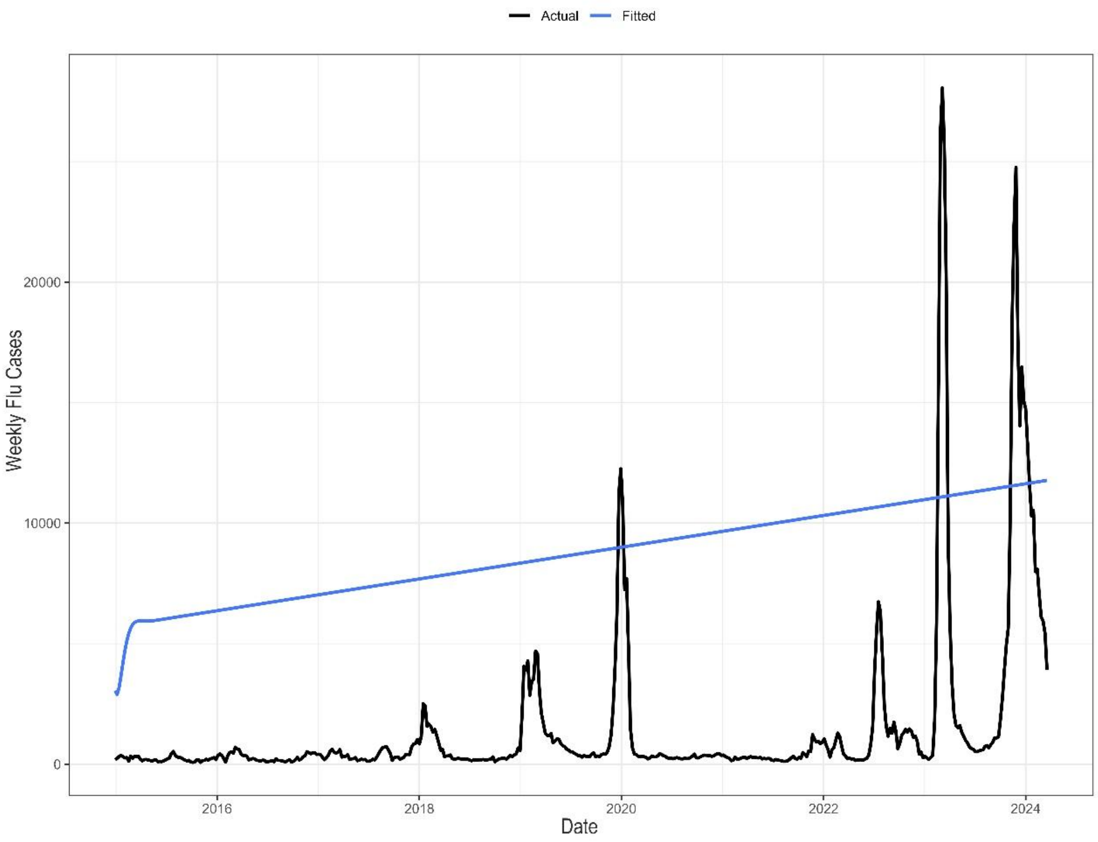

Figure S3. Fitting performance of the ARIMA model for influenza incidence.

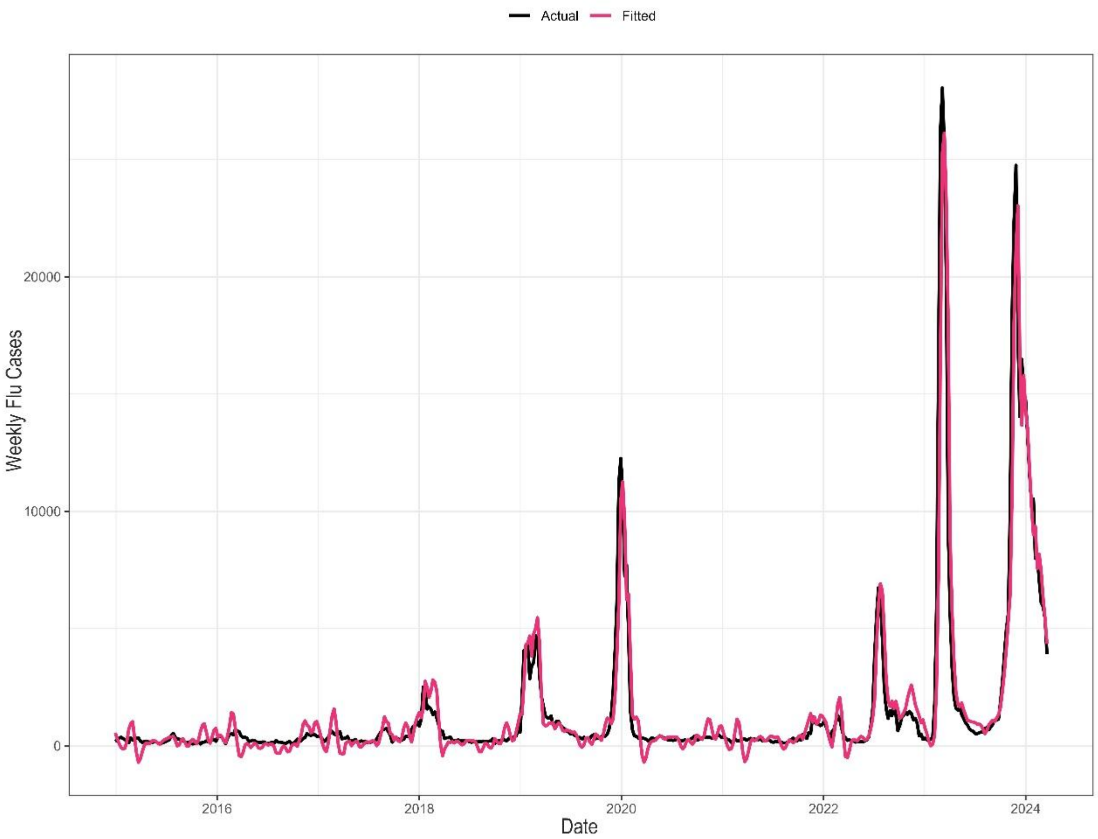

Figure S4. Fitting performance of the Prophet model for influenza incidence.

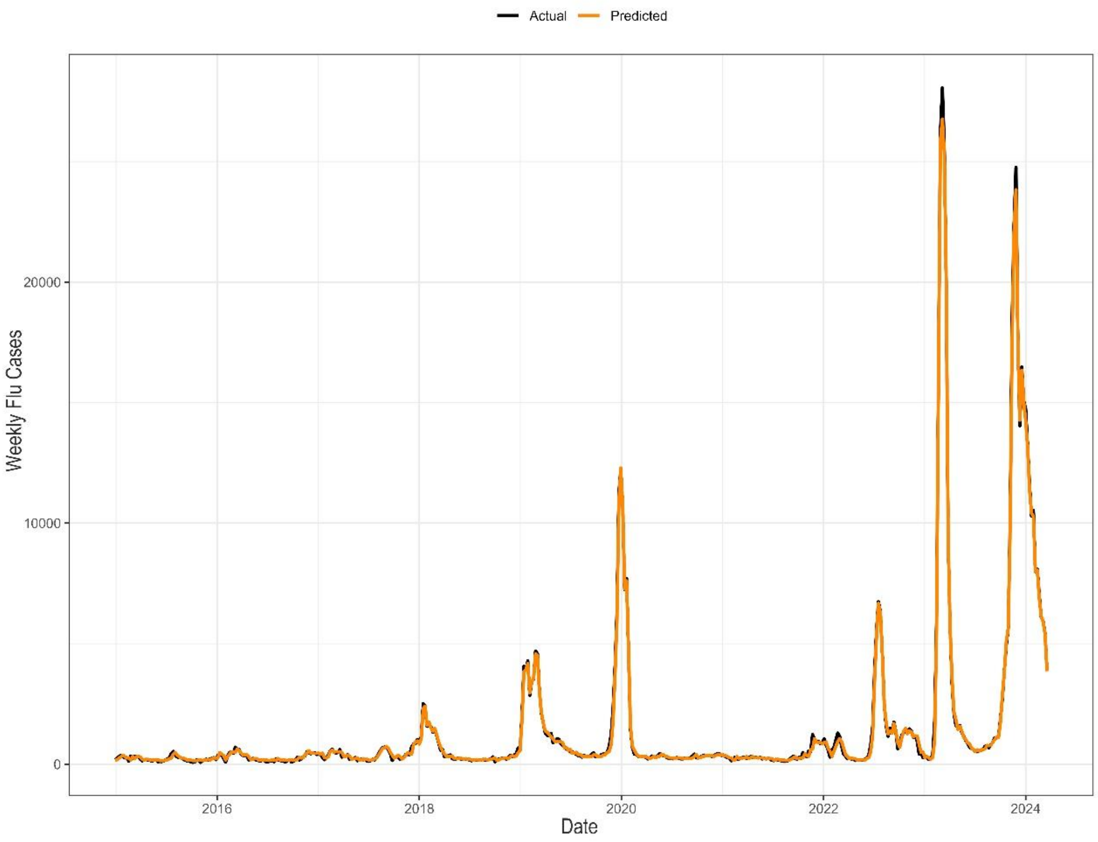

Figure S5. Fitting performance of the XGBoost model for influenza incidence.

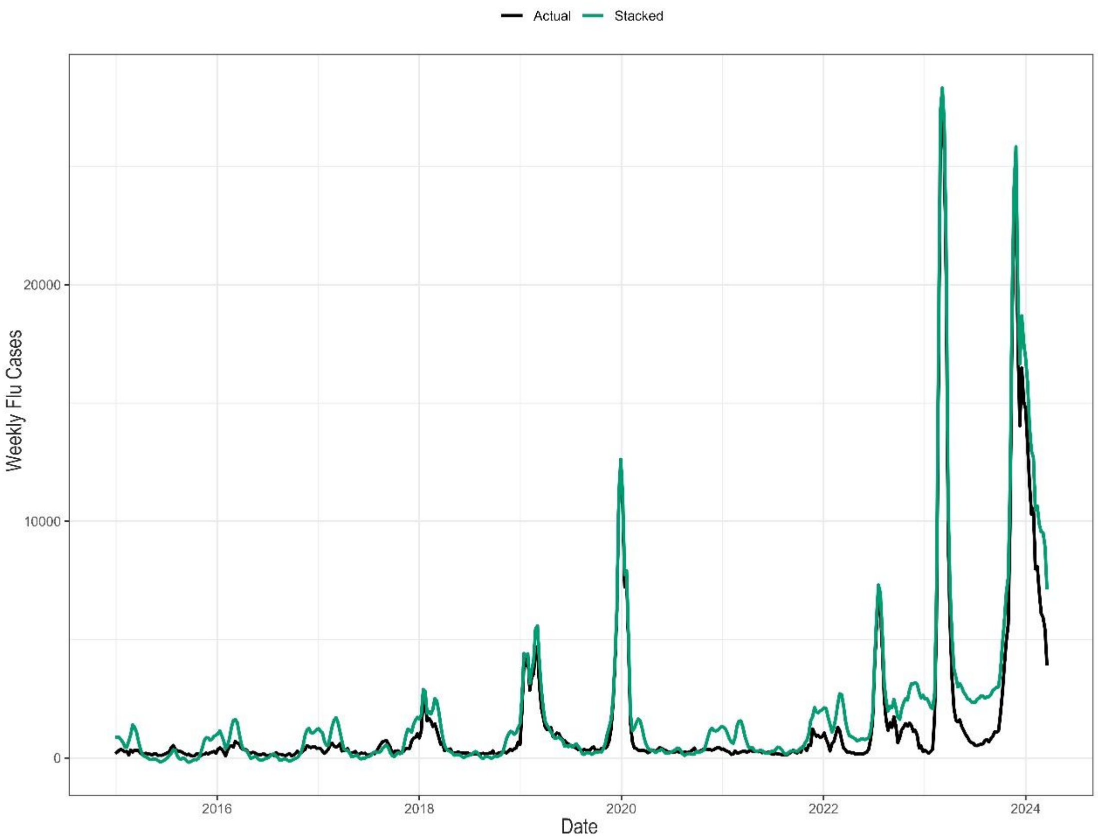

Figure S6. Fitting performance of the stacked ensemble model for influenza incidence.

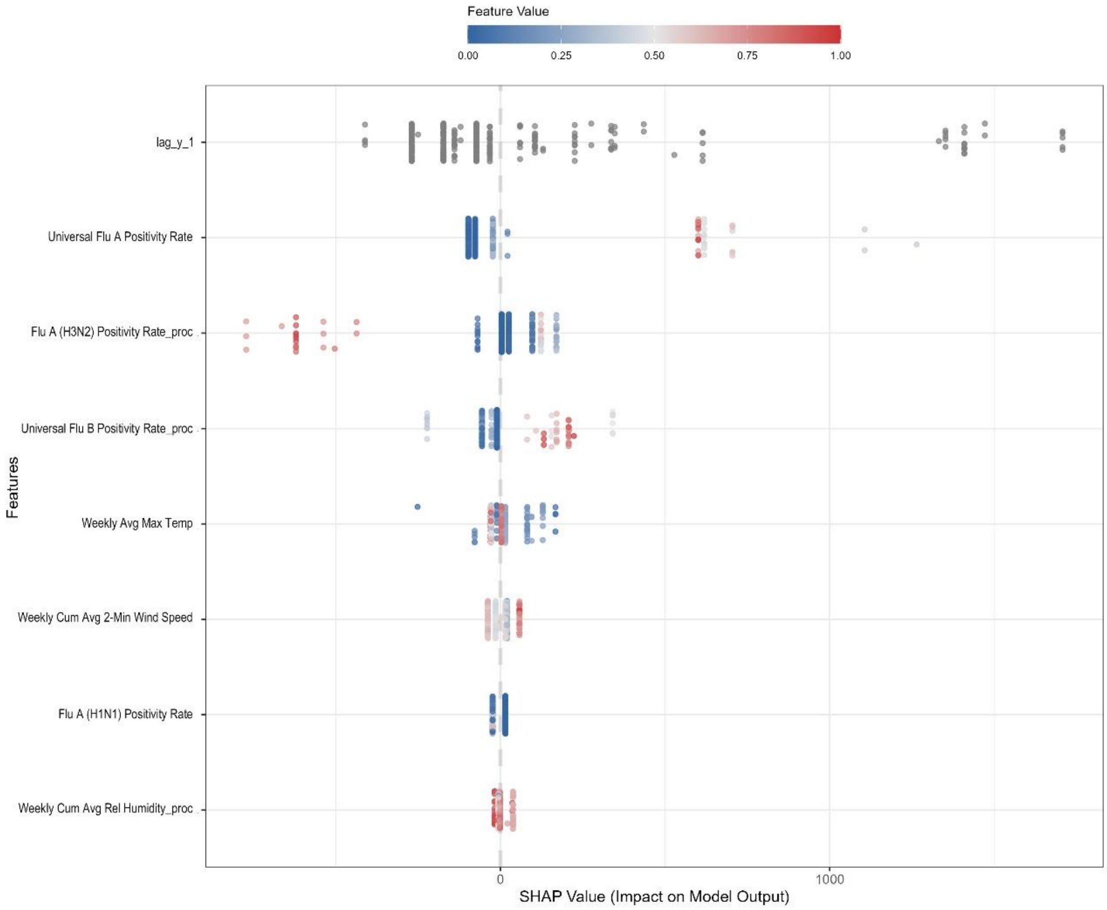

Figure S7. SHAP dependence plots for key features in the stacked ensemble model.

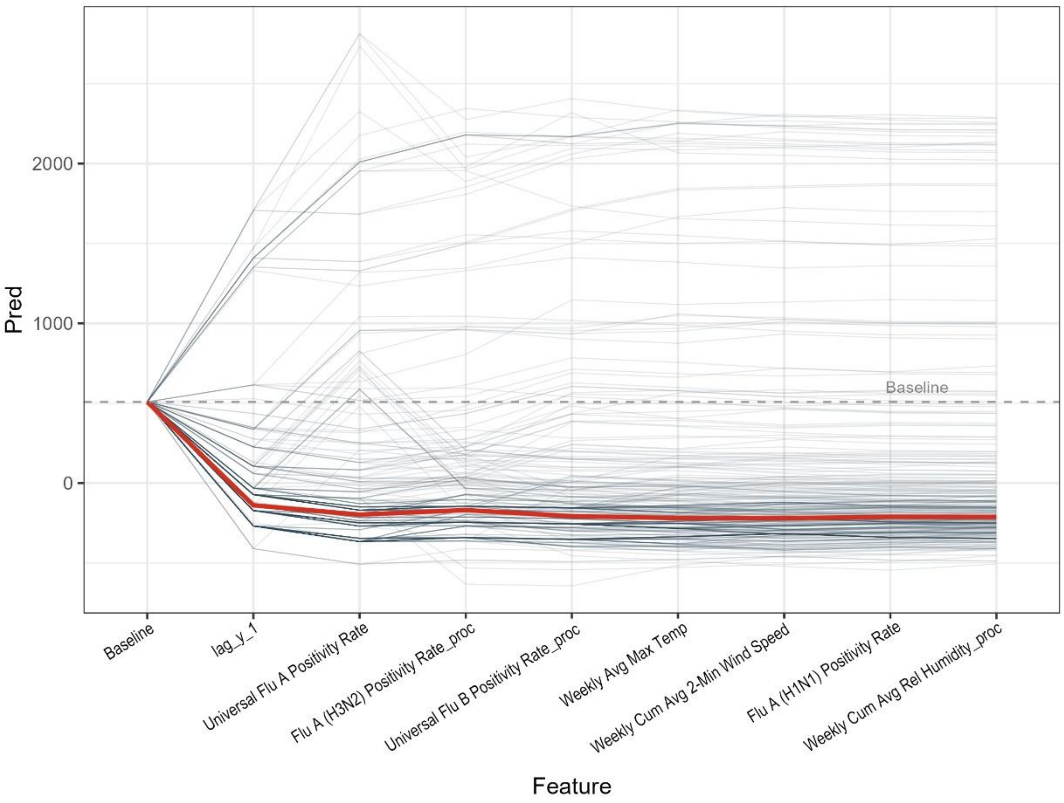

Figure S8. Decision paths of feature contributions in the stacked ensemble model.

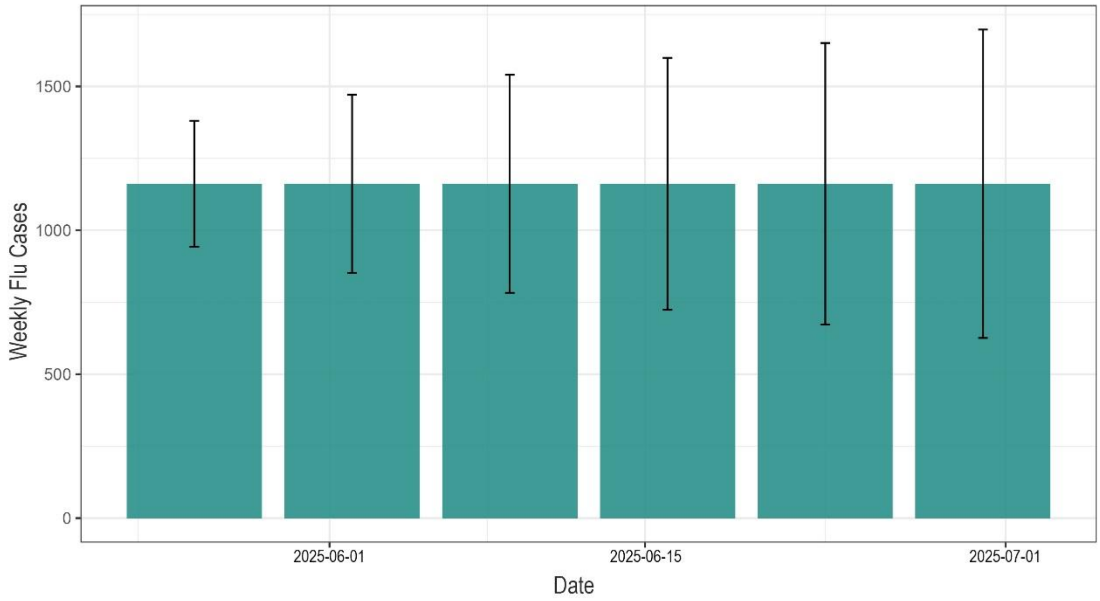

Figure S9. Enlarged view of the six-week forecast of influenza incidence.

Table S1. Missingness proportion for study variables.

Variable (manuscript-ready)	Missingness proportion
Epidemiological week	0
Influenza A overall positivity rate	0.0957642726
Influenza A (H3N2) positivity rate	0.0957642726
Influenza A (H1N1) positivity rate	0.0957642726
Influenza B overall positivity rate	0.0957642726
Influenza B (Yamagata lineage) positivity rate	0.0976058932
Influenza B (Victoria lineage) positivity rate	0.0976058932
SARS-CoV-2 positivity rate	1
Number of positive tests for influenza A overall	0.0957642726
Number of negative tests for influenza A overall	0.0957642726
Number of untested samples for influenza A overall	0.0957642726
Number of positive tests for influenza A (H3N2)	0.0957642726
Number of negative tests for influenza A (H3N2)	0.0957642726
Number of untested samples for influenza A (H3N2)	0.0957642726
Number of positive tests for influenza A (H1N1)	0.0957642726
Number of negative tests for influenza A (H1N1)	0.0957642726
Number of untested samples for influenza A (H1N1)	0.0957642726
Number of positive tests for influenza B overall	0.0957642726
Number of negative tests for influenza B overall	0.0957642726
Number of untested samples for influenza B overall	0.0957642726
Number of positive tests for influenza B (Yamagata lineage)	0.0957642726
Number of negative tests for influenza B (Yamagata lineage)	0.0957642726
Number of untested samples for influenza B (Yamagata lineage)	0.0957642726
Number of positive tests for influenza B (Victoria lineage)	0.0957642726
Number of negative tests for influenza B (Victoria lineage)	0.0957642726
Number of untested samples for influenza B (Victoria lineage)	0.0957642726
Number of positive tests for SARS-CoV-2	0.0957642726
Number of negative tests for SARS-CoV-2	0.0957642726
Number of untested samples for SARS-CoV-2	0.0957642726
Total number of samples	0.0957642726
Number of valid tests	0.0957642726
Weekly mean of average temperature	0.2283609576
Weekly cumulative average temperature	0.2283609576
Weekly mean of maximum temperature	0.2283609576
Weekly cumulative maximum temperature	0.2283609576
Weekly mean of minimum temperature	0.2283609576
Weekly cumulative minimum temperature	0.2283609576
Weekly mean of relative humidity	0.2283609576

Variable (manuscript-ready)	Missingness proportion
Weekly cumulative relative humidity	0.2283609576
Weekly mean of 2-minute wind speed	0.2283609576
Weekly cumulative 2-minute wind speed	0.2283609576
Weekly mean precipitation (20:00–20:00)	0.2283609576
Weekly cumulative precipitation (20:00–20:00)	0.2283609576
Number of influenza cases	0.0055248619

Table S2. Performance metrics of the candidate models on the training set.

**Model**	**RMSE**	**MAE**	**MAPE**	**R^2**	**Adjusted R^2**
ARIMA	7972.82	7688.95	2437.70	-3.66	-3.67
Prophet	2912.69	1613.57	284.55	0.38	0.38
XGBoost	140.73	76.23	16.19	0.99	0.99
Stacked	923.12	621.99	118.04	0.94	0.94
